# A randomized, placebo-controlled trial of d-cycloserine for the enhancement of social skills training in autism spectrum disorders

**DOI:** 10.1186/s13229-015-0062-8

**Published:** 2016-01-14

**Authors:** Noha F. Minshawi, Logan K. Wink, Rebecca Shaffer, Martin H. Plawecki, David J. Posey, Hai Liu, Sarah Hurwitz, Christopher J. McDougle, Naomi B. Swiezy, Craig A. Erickson

**Affiliations:** Christian Sarkine Autism Treatment Center, Riley Hospital for Children at Indiana University Health, Indiana University School of Medicine Department of Psychiatry, Indianapolis, IN USA; Cincinnati Children’s Hospital Medical Center, University of Cincinnati College of Medicine, 3333 Burnet Avenue MLC 4002, Cincinnati, OH 45229 USA; David J. Posey, M.D., LLC, Indianapolis, IN USA; Department of Biostatistics, Indiana University School of Medicine, Indianapolis, IN USA; Indiana University School of Education, Bloomington, IN USA; Lurie Center for Autism, Departments of Psychiatry and Pediatrics, Massachusetts General Hospital and MassGeneral Hospital for Children, Harvard Medical School, Boston, MA USA

**Keywords:** d-cycloserine, Autism spectrum disorders, Social skills training, Social deficits

## Abstract

**Background:**

Researchers have demonstrated that d-cycloserine (DCS) can enhance the effects of behavioral interventions in adults with anxiety and enhances prosocial behavior in animal models of autism spectrum disorders (ASD). This study extended upon this background by combining DCS with behavioral social skills therapy in youth with ASD to assess its impact on the core social deficits of ASD. We hypothesized that DCS used in combination with social skills training would enhance the acquisition of social skills in children with ASD.

**Methods:**

A 10-week, double-blind, placebo-controlled trial of DCS (50 mg) given 30 min prior to weekly group social skills training was conducted at two sites. Children with ASD were randomized to receive 10 weeks (10 doses) of DCS or placebo in a 1:1 ratio.

**Results:**

No statistically significant difference attributable to drug treatment was observed in the change scores for the primary outcome measure, the Social Responsiveness Scale (SRS), total score (*p* = 0.45), or on secondary outcome measures.

**Conclusions:**

The results of this trial demonstrated no drug-related short-term improvement on the primary outcome measure, or any of the secondary outcome measures. However, an overall significant improvement in SRS total raw score was observed from baseline to end of treatment for the entire group of children with ASD. This suggests a need to further study the efficacy of the social skills training protocol. Limitations to the current study and areas for future research are discussed.

**Trial registration:**

ClinicalTrials.govNCT01086475

## Background

Autism spectrum disorders (ASD), including autistic disorder, Asperger’s disorder, and pervasive developmental disorder not otherwise specified (PDD-NOS), have received increasing attention from researchers, clinicians, and the public since autism was first described by Leo Kanner in 1943 [1]. The diagnosis of ASD is characterized by core social and communication deficits, as well as restricted, repetitive behaviors. In recent years, the rates of ASD have escalated, with the most recent Centers for Disease Control and Prevention data estimating prevalence at 1 in 68 children in the USA [2]. While some successful pharmacological and behavioral interventions have been identified for the treatment of hyperactivity/inattention and irritability associated with ASD [3, 4], little progress has been made in the effective treatment of primary social and communication deficits. The limited success of clinical trials targeting core social impairment in ASD is likely in part due to the heterogeneity of ASD, difficulty quantitatively tracking treatment response, and high placebo response rates [5]. Regardless, the lack of viable treatments is particularly concerning given that pervasive social impairment in ASD can limit lifelong functioning and independence [6].

Research in psychiatric disorders has led to some advances in ASD research. Specifically, a parallel is frequently drawn between schizophrenia and ASD due to similarity between the negative symptoms of schizophrenia and social withdrawal seen in ASD, as well as the implication of glutamate dysregulation in both disorders [7]. Consequently, several targeted treatment trials in both ASD and schizophrenia have focused on modulating glutamate neurotransmission [5, 8]. d-cycloserine (DCS), a partial agonist of the *N*-methyl-d-aspartate (NMDA) glutamate receptor and a Food and Drug Administration-approved treatment for tuberculosis, has been researched for treatment of negative symptoms of schizophrenia with mixed results [8–12]. In ASD, a single-blind pilot study of DCS in children and adults (mean age of 10 years) found that DCS was associated with a clinically significant reduction in social withdrawal and increase in social responsiveness compared to a placebo control [13]. However, a double-blind, placebo-controlled trial of daily dosing of DCS in 88 children with ASD found no significant difference in measures of social withdrawal or global severity ratings during 8 weeks of daily treatment [14].

Glutamatergic neurotransmission has also been of interest in the treatment of anxiety disorders [15, 16]. A growing body of preclinical and clinical research has demonstrated the ability of DCS to enhance learning in the treatment of anxiety symptoms [17]. The mechanism believed to be responsible for this effect is the enhancement of learned extinction of fear responses via combination cognitive behavior therapy (CBT) and DCS treatment [17, 18]. Results have shown that DCS plays an augmentative role in the learning that takes place during CBT and therefore leads to greater success than when CBT is used alone.

The promising results from anxiety studies focused on extinction-based learning, as well as the role of DCS in other forms of learning, have subsequently led to the investigation of the role of DCS in the enhancement of social learning in animal models of ASD. Modi and Young [19] demonstrated that DCS combined with social learning paradigms in mice increased prosocial bonding and partner selection. This model of social learning may be similar to the social learning that takes place during social interactions and behavioral skills training in individuals with ASD. However, no studies of combined DCS plus nondrug therapy have been published in children with ASD. Based on this background, we investigated DCS treatment in combination with behavior therapy in youth with ASD. We hypothesized that DCS used in combination with social skills training utilizing the technology of applied behavior analysis (ABA), the most empirically supported behavioral intervention for ASD [20], would enhance the acquisition of social skills in children with ASD. The social skills training curriculum involved a combination of didactic instruction in the form of social stories [21, 22], discussions, discrimination training tasks, as well as performance and feedback-based instruction, such as role playing and modeling of skills. Typically developing peers were also incorporated as models in all groups. Social skills training that includes the use of typically developing peers as models and agents of intervention has been shown to increase the social interactions of children with ASD [23, 24]. The social skills group curriculum being utilized in this study was previously investigated via a pilot feasibility study with eight children with ASD and four typically developing peers. This pilot study used the Triad social skills assessment (TSSA) [25], social skills measure, as the primary outcome and evaluated 8 h of intervention. Children who participated in the social skills training demonstrated a significant improvement in overall social skill ability on the TSSA at post-test (*p* < 0.05). The children with ASD showed significant improvement in understanding emotions, initiating interactions, responding to interactions, and general social competency [26]. We additionally hypothesized that children treated with DCS would show greater improvement in social functioning from social skills training than those taking placebo.

## Methods

### Study design

A 10-week, double-blind, placebo-controlled trial of low dose (50 mg) DCS given 30 min prior to weekly group social skills training was conducted at two sites, Indiana University School of Medicine and Cincinnati Children’s Hospital Medical Center. Children with ASD were randomized to receive 10 weeks (10 doses) of DCS or placebo in a 1:1 ratio. All children received 10 weeks of manualized social skills training. Children were further divided into two age groups, 5–7 and 8–11 years, for the purposes of keeping social skills groups more homogeneous. Each social skills group included up to four children with ASD and two typically developing peer models (TPs) in the same age group. The TPs participated in all group activities but did not take DCS or placebo. Adverse events (AEs) and interval history were collected prior to dosing, and outcome measures were administered at baseline, week 6, and week 11. This trial was approved by the Institutional Review Board at each site.

### Participants

Sixty-seven children with ASD ages 5–11 years participated in the study along with 34 typically developing, same-aged children who served as TPs. One subject with ASD was excluded from the analyses due to early dropout prior to taking the study drug. Participants were recruited from academic autism treatment centers, local schools, and community organizations. Written informed consent was obtained from legal guardians, and assent was obtained when participants were able. Diagnosis of ASD was made through administration of the Autism Diagnostic Observation Schedule [27, 28], Autism Diagnostic Interview-Revised [29], and clinical interview using the *Diagnostic and Statistical Manual of Mental Disorders*, *Fourth Edition*, *Text Revision* (DSM-IV-TR) [30] criteria for autistic disorder, Asperger’s disorder, or pervasive developmental disorder not otherwise specified (PDD-NOS).

Subjects with ASD were required to have an intellectual quotient greater than 70 on the *Stanford*-*Binet 5th Edition* [31] (SB-V) and a communication standard score greater than 70 on the *Vineland Adaptive Behavior Scale 2nd Edition* (VABS-II) [32] survey edition. These criteria were included to ensure that participants did not have cognitive or language deficits that could interfere with their ability to participate in group social skills training. Additional inclusion criteria included a Triad social skills assessment (TSSA) [25] score of 70 % or less on both parent questionnaire and child assessment, significant social impairment as measured by a *T* score of 60 or greater on the Social Responsiveness Scale (SRS) [33] and Clinical Global Impression-Severity (CGI-S) scale score of at least four (moderately ill). The CGI-S is a clinician-rated global assessment of symptom severity. The CGI-S item is rated on a scale from 1 to 7 (1 = normal, not at all ill; 2 = borderline ill; 3 = mildly ill; 4 = moderately ill; 5 = markedly ill; 6 = severely ill; 7 = among the most extremely ill patients). Rater training was conducted with gold standard vignettes and inter-rater reliability of 80 % or greater was established.

Study participants were required to remain on stable psychotropic medication dosing targeting symptoms associated with ASD (e.g., insomnia, inattention, hyperactivity, anxiety, irritability) for a minimum of 2 weeks (with the exception of 4 weeks for fluoxetine) prior to randomization. Potential participants were excluded if they were taking more than two psychotropic medications or if they were currently taking a glutamatergic modulator (e.g., riluzole, memantine, acamprosate, topiramate, amantadine). In addition, concomitant psychosocial treatments could not include group social skills training outside of the study, and all the therapies were required to have been stable for at least 90 days prior to randomization.

The TPs were screened with the Child Symptom Inventory-4 [34] to ensure that they did not have a history of psychiatric symptoms that were currently affecting social skills (e.g., attention-deficit/hyperactivity disorder, oppositional defiant disorder, schizophrenia, ASD, social anxiety disorder, and major depression). The child’s appropriateness for inclusion in the social skills groups (e.g., absence of social, behavioral, or language problems) was also assessed by a trained clinician. Parents of TPs provided informed consent and TPs provided assent.

### Social skills training

Social skills groups were conducted following a manualized curriculum adapted for use in the present study. The curriculum utilized ABA-based methodologies, including shaping, incidental teaching, positive reinforcement, and visual schedules, as well as social stories and weekly parent-mediated homework assignments.

Sessions focused on a specific social skill topic each week: greetings, emotions, conversations, review, and saying good-bye. The curriculum included a variety of techniques that have been shown to help children with ASD learn social skills. A sample curriculum is provided in Table 1. Specific techniques included sorting examples of appropriate and inappropriate behaviors, reading social stories, engaging in role-plays, labeling flash cards, engaging in art activities, and playing various games. Minor modifications were made to curriculum based on the age group (5–7 or 8–11 years old) to enhance understanding and developmental appropriateness. Social groups were facilitated by masters or doctoral-level clinicians with expertise in ASD and ABA. Therapists were trained in the curriculum by a lead therapist (the study’s principle investigator), and significant therapist overlap occurred both within and across study sites to address consistency in therapist techniques. However, treatment fidelity data was not collected.

**Table 1:** Sample characteristics at baseline

Characteristics	DCS (n=34)	Placebo (n=33)	p-value
Demographics			
Age (years), mean (SD)	8.38 (1.93)	8.25 (1.73)	.76
Sex, n (%) male	28 (82.35)	27 (81.82)	.95
Clinical Variables			
Stanford-Binet V, mean (SD)			
Full Scale IQ	92.42 (17.76)	87.30 (15.74)	.22
NonVerbal IQ	95.15 (18.03)	90.82 (15.19)	.30
Verbal IQ	90.85 (18.97)	85.45 (16.83)	.23
VABS-II Expressive Language subscale standard score, mean (SD)	87.38 (13.36)	84.55 (14.94)	.42
Clinical Global Impression-Severity	4.03 (0.18)	4.06 (0.24)	.58
Diagnosis, n (%)			
PDD-NOS	12 (35.29)	15 (45.45)	.40
Autistic Disorder	3 (8.82)	5 (15.15)	.48^a^
Asperger’s Disorder	19 (55.88)	13 (39.39)	.18
Concomitant medications, n (%)			
Antipsychotics	8 (23.53)	8 (24.24)	.95
Alpha-2 Agonists	6 (17.65)	8 (24.24)	.51
Stimulants	14 (41.18)	11 (33.33)	.51
Sleep Aids	9 (26.47)	7 (21.21)	.61
Mood Stabilizers	1 (2.94)	2 (6.06)	.61^a^
Glutamatergic Modulators	1 (2.94)	0 (0.00)	1.00^a^
Other	3 (8.82)	1 (3.03)	.61^a^
Concomitant treatment, n (%)			
Speech Therapy	19 (55.88)	16 (51.52)	.72
Occupational Therapy	12 (35.29)	15 (45.45)	.40
Behavioral Therapy	9 (26.47)	9 (27.27)	.94
Other Psychotherapy	1 (2.94)	2 (6.06)	.61^a^
Physical Therapy	0 (0.00)	2 (6.06)	.24^a^
Social Skills Training	3 (8.82)	2 (6.06)	1.00^a^
Music Therapy	0 (0.00)	1 (3.03)	.49^a^
Other Treatments	3 (8.82)	0 (0.00)	.24^a^

^a^: Fisher’s Exact Test

The TPs assisted in modeling and reinforcing appropriate behavior during each group session. The TPs were recruited from local school districts and via media advertisements. Prior to the start of social skills training, TPs were educated in a separate session. An introduction to behaviors associated with ASD was presented, along with an overview of the social skills curriculum and weekly schedule. In addition, TPs engaged in role play with the clinicians to practice appropriate skills and corrective feedback was provided. A social story on ASD was also provided for the TPs to review at home with their parents prior to the first social skills group.

### Primary outcome

The primary outcome measure of the ASD phenotype and social interactions in participants with ASD was the parent-rated SRS total raw score. The SRS is a standardized, 65-item measure of the core symptoms of ASD where each item is scored on a 4-point scale, which has been used extensively in ASD research [35–38]. The SRS was administered at screen, baseline, week 6 (after 5 weeks of SST), and at week 11 (after 10 weeks of social skills training).

### Secondary outcomes

Several secondary outcome measures were included to capture different aspects of ASD that could be affected by the proposed treatment. When available, SRS data was collected from teachers of the subjects with ASD at baseline, week 6, and week 11. Additionally, all participants were evaluated using the VABS-II, Aberrant Behavior Checklist (ABC) [39], Clinical Global Impression Improvement Scale (CGI-I), and the TSSA, at baseline and week 11.

The adaptive functioning of subjects was evaluated at baseline and week 11 using the VABS-II. The VABS-II assesses adaptive functioning in four domains: communication, daily living skills, socialization, and motor skills. Administered via semi-structured interview with parents or caregiver, the VABS-II provides a measure of overall functioning of children and adults. The VABS-II is a standardized, norm-referenced assessment that is used extensively in individuals with ASD [40, 41].

The ABC was collected at baseline, week 6 and week 11 to assess the impact of the treatment on symptoms relevant to ASD. The ABC is a 58-item parent questionnaire with five subscales derived by factor analysis: irritability, social withdrawal, stereotypy, hyperactivity, and inappropriate speech. The ABC has been extensively used in psychopharmacological studies of ASD [42]. When available, teachers of the subjects with ASD were also asked to complete the ABC at the same time points.

The CGI-I was utilized as a clinician-rated dichotomous outcome measure to assess response to treatment. A trained clinician blind to treatment assignment rated the CGI-S at baseline and the CGI-I at each visit following randomization. Factors included in rating the CGI-I included parent report, parent-rated measures, teacher-rated measures, and clinician-rated measures. The CGI-I provides a qualitative measure of treatment response through a rating from 1 to 7 (1 = very much improved; 2 = much improved; 3 = minimally improved; 4 = no change; 5 = minimally worse; 6 = much worse; 7 = very much worse). Rater training was conducted with gold standard vignettes and inter-rater reliability of 80 % or greater was established. At the end of treatment, subjects with a CGI-I of “1” or “2” were categorized as responding to the treatment and subjects with CGI-I scores of “3” or higher were categorized as nonresponders.

To assess the impact of the treatment on social skills and social knowledge, the TSSA was administered to the subjects and their parents at baseline and week 11. The TSSA is a criterion-based assessment that addresses three components of social knowledge and skills: cognitive (ability to problem-solve interpersonal conflicts), behavioral (ability to initiate and maintain interactions and respond appropriately to others), and affective (ability to understand emotions). The TSSA has been used as a supplemental descriptive measure of social skills [43], as well as in treatment planning [44]. Other outcome measures collected included eye tracking data and direct behavioral observations (not reported in the present paper).

Finally, monitoring for AEs was completed at each visit for subjects with ASD. The site physician kept a log of AEs that included the date of onset, date of resolution, severity, and relationship to study intervention (e.g., definite, probable, possible, remote, or none).

Parental satisfaction with the study and social skills training group was measured via questionnaire at the end of the study (week 11). Parents were asked to answer the question, “participating in this group was worthwhile for my family”, on a five-point Likert scale with “1” being anchored to “strongly disagree” and “5” being “strongly agree”.

### Statistical analysis

Study participants’ demographic and clinical characteristics were summarized and compared between the DCS and placebo groups at baseline using two-sample *t* tests for continuous variables and Fisher’s exact tests for categorical variables. The change scores of the primary outcome variables (SRS total score and subscales) from baseline to 11-week follow-up were also compared between the two treatment groups using *t* tests. Similar analyses were conducted for the secondary outcomes including VABS-II total score and subscales, ABC subscales, and TSSA parent report. In addition, a linear mixed-effects modeling was used to further test the treatment effect over time using longitudinal SRS total scores measured at baseline, 6-week and 11-week visits. Responder analysis (responders were defined as “much improved” or “very much improved” for CGI-I at 11-week follow-up) was conducted using chi-square test. AEs during the treatment period were also analyzed. All analyses were performed using SAS version 9.2.

## Results

Thirty-four participants were randomized to the DCS treatment group, and 33 were randomized to the placebo group. One subject who was randomized to the placebo group dropped out of the study before taking any medication and subsequently was excluded from the analysis. Comparisons between the two groups showed no statistically significant difference in age, sex, SB-V scores, the VABS-II Expressive Language subscale, the CGI-S, concomitant medications, or concomitant therapy treatments at baseline (Table 1). Therefore, no potential confounders were adjusted for as covariates in all subsequent analyses. Furthermore, no significant differences were noted between the two sites (Cincinnati and Indiana University) on demographic variables (Table 2).

**Table 2:** Sample characteristics at enrollment across sites

Characteristics	Cincinnati (n=15)	Indiana University (n=52)	p-value
Demographics			
Age (years), mean (SD)	8.56 (1.77)	8.24 (1.84)	.56
Sex, n (%) male	11 (73.33)	44 (84.62)	.44^a^
Group, n (%) Cycloserine	8 (53.33)	26 (50.00)	.82
Clinical Variables			
Stanford-Binet V, mean (SD)			
Full Scale IQ	87.86 (13.78)	90.40 (17.67)	.62
NonVerbal IQ	92.57 (15.36)	93.10 (17.17)	.92
Verbal IQ	85.29 (14.19)	88.92 (18.94)	.51
VABS-II Expressive Language subscale standard score, mean (SD)	84.67 (9.76)	86.37 (15.21)	.68
Clinical Global Impression-Severity	4.00 (0.00)	4.06 (0.24)	.38
Diagnosis, n (%)			
PDD-NOS	9 (60.00)	18 (34.62)	.08
Autistic Disorder	4 (26.67)	4 (7.69)	.07^a^
Asperger’s Disorder	2 (13.33)	30 (57.69)	.003^a*^
Concomitant medications, n (%)			
Antipsychotics	2 (13.33)	14 (26.92)	.49^a^
Alpha-2 Agonists	3 (20.00)	11 (21.15)	1.00^a^
Stimulants	8 (53.33)	17 (32.69)	.15
Sleep Aids	3 (20.00)	13 (25.00)	1.00^a^
Mood Stabilizers	0 (0.00)	3 (5.77)	1.00^a^
Glutamatergic Modulators	0 (0.00)	1 (1.92)	1.00^a^
Other	1 (6.67)	3 (5.77)	1.00^a^
Concomitant treatment, n (%)			
Speech Therapy	6 (40.00)	30 (57.69)	.23
Occupational Therapy	6 (40.00)	21 (40.38)	.98
Behavioral Therapy	2 (13.33)	16 (30.77)	.32^a^
Other Psychotherapy	0 (0.00)	3 (5.77)	1.00^a^
Physical Therapy	1 (6.67)	1 (1.92)	.40^a^
Social Skills Training	1 (6.67)	4 (7.69)	1.00^a^
Music Therapy	1 (6.67)	0 (0.00)	.22^a^
Other Treatments	1 (6.67)	2 (3.85)	.54^a^

^a^: Fisher’s Exact Test

No statistically significant difference attributable to drug treatment was observed in the change scores for the SRS total score (*p* = 0.45). The SRS subscale scores were evaluated as an exploratory analysis and were also not statistically significant. Additionally, no significant differences that were identified between groups in the change scores for the secondary outcome measures were identified (Table 3). In addition, teacher-rated ABC data was returned for 23.5 % of the DCS group, and 30.3 % of the placebo group with no significant difference noted for any of the ABC subscales (irritability *p* = 0.623, social withdrawal *p* = 0.845, stereotypy *p* = 0.434, hyperactivity *p* = 0.833, and inappropriate speech *p* = 0.959) between groups. Teacher-rated SRS data was available for 26.4 % of the DCS group and 27.2 % of the placebo group, and again, no significant difference was found between groups (*p* = 0.59). Finally, at week 11, parental satisfaction was found to be high across both groups (96 % and 88 % for DCS and placebo groups, respectively, rated satisfaction as a 4 or 5 on the five-point Likert scale). A Fisher’s exact test demonstrated no significant difference in the level of parental satisfaction (*p* = 0.33).

**Table 3:** Baseline, Week 11, and change in primary and secondary outcome measures

Clinical Outcome	DCS (n=34)			Placebo (n=33)				
	Mean (SD)			Mean (SD)				
	Baseline	11-week	Change	Baseline	11-week	Change	Difference in Change Scores (95% CI)	P-value
Primary outcome								
Social Responsiveness Scale (SRS) Parent Raw Scores								
Social awareness	12.82 (3.43)	12.15 (2.73)	-0.76 (2.60)	13.82 (3.59)	12.94 (2.67)	-1.00 (3.12)	0.24 (-1.19 to 1.67)	.74
Social cognition	19.18 (5.37)	17.21 (6.01)	-1.94 (4.41)	20.88 (4.34)	18.06 (4.24)	-2.68 (3.74)	0.74 (-1.31 to 2.79)	.47
Social communication	34.94 (8.65)	30.00 (8.55)	-4.91 (6.49)	37.97 (7.84)	32.03 (7.71)	-6.19 (8.40)	1.28 (-2.45 to 5.02)	.49
Social motivation	16.06 (5.84)	13.18 (5.01)	-2.76 (3.95)	16.33 (5.34)	13.39 (5.17)	-3.06 (4.40)	0.31 (-1.78 to 2.40)	.54
Autistic mannerisms	19.44 (6.10)	16.42 (6.03)	-2.82 (5.24)	21.42 (6.27)	17.65 (5.36)	-4.10 (6.38)	1.28 (-1.63 to 4.19)	.38
SRS Parent Total Score	102.35 (25.09)	88.67 (22.74)	-13.39 (16.81)	110.33 (20.43)	94.00 (19.31)	-17.00 (21.33)	3.61 (-5.96 to 13.17)	.45
Secondary outcomes								
VABS-II raw scores								
Communication	152.56 (17.05)	156.47 (17.85)	3.63 (8.26)	147.06 (22.18)	154.58 (21.93)	6.77 (11.15)	-3.15 (-8.08 to 1.79)	.21
Daily living skills	231.62 (26.08)	239.56 (26.99)	7.06 (14.72)	229.67 (35.43)	239.29 (35.76)	8.45 (16.82)	-1.39 (-9.35 to 6.57)	.73
Socialization	113.26 (24.98)	126.53 (27.54)	12.34 (20.00)	103.97 (22.14)	117.35 (27.94)	12.10 (22.28)	0.25 (-10.41 to 10.91)	.96
Motor skills	141.21 (7.21)	143.59 (5.42)	2.22 (5.70)	141.27 (9.50)	142.19 (9.59)	0.19 (3.81)	2.03 (-0.41 to 4.46)	.10
Maladaptive behavior	16.65 (6.87)	15.88 (7.12)	-0.59 (5.92)	18.55 (7.42)	16.42 (5.59)	-2.03 (5.91)	1.44 (-1.54 to 4.42)	.34
Total	655.29 (59.88)	682.03 (64.28)	24.66 (37.56)	640.52 (78.65)	669.84 (83.74)	25.48 (41.11)	-0.83 (-20.66 to 19.00)	.93
ABC parent score								
Irritability	11.06 (8.05)	9.12 (6.63)	-1.55 (6.11)	12.67 (8.96)	11.06 (7.42)	-1.23 (6.09)	-0.32 (-3.37 to 2.73)	.83
Social Withdrawal	10.29 (8.30)	8.06 (7.61)	-2.39 (4.71)	10.39 (8.33)	8.81 (6.41)	-1.71 (7.04)	-0.68 (-3.71 to 2.34)	.65
Stereotypy	4.94 (4.21)	4.64 (4.89)	-0.33 (2.79)	4.64 (4.32)	4.32 (3.75)	-0.45 (4.60)	0.12 (-1.81 to 2.04)	.90
Hyperactivity	18.82 (11.26)	17.30 (12.23)	-1.55 (7.37)	19.00 (9.18)	16.87 (9.49)	-2.03 (9.39)	0.49 (-3.72 to 4.69)	.82
Inappropriate speech	3.91 (3.04)	3.24 (2.26)	-0.73 (2.59)	4.36 (2.85)	4.68 (3.29)	0.35 (2.67)	-1.08 (-2.40 to 0.23)	.10
TSSA parent	53.74 (8.61)	57.84 (9.11)	3.88 (3.49)	48.79 (8.91)	54.34 (10.37)	5.83 (9.70)	-1.95 (-6.87 to 2.97)	.43

**Fig. 1:**
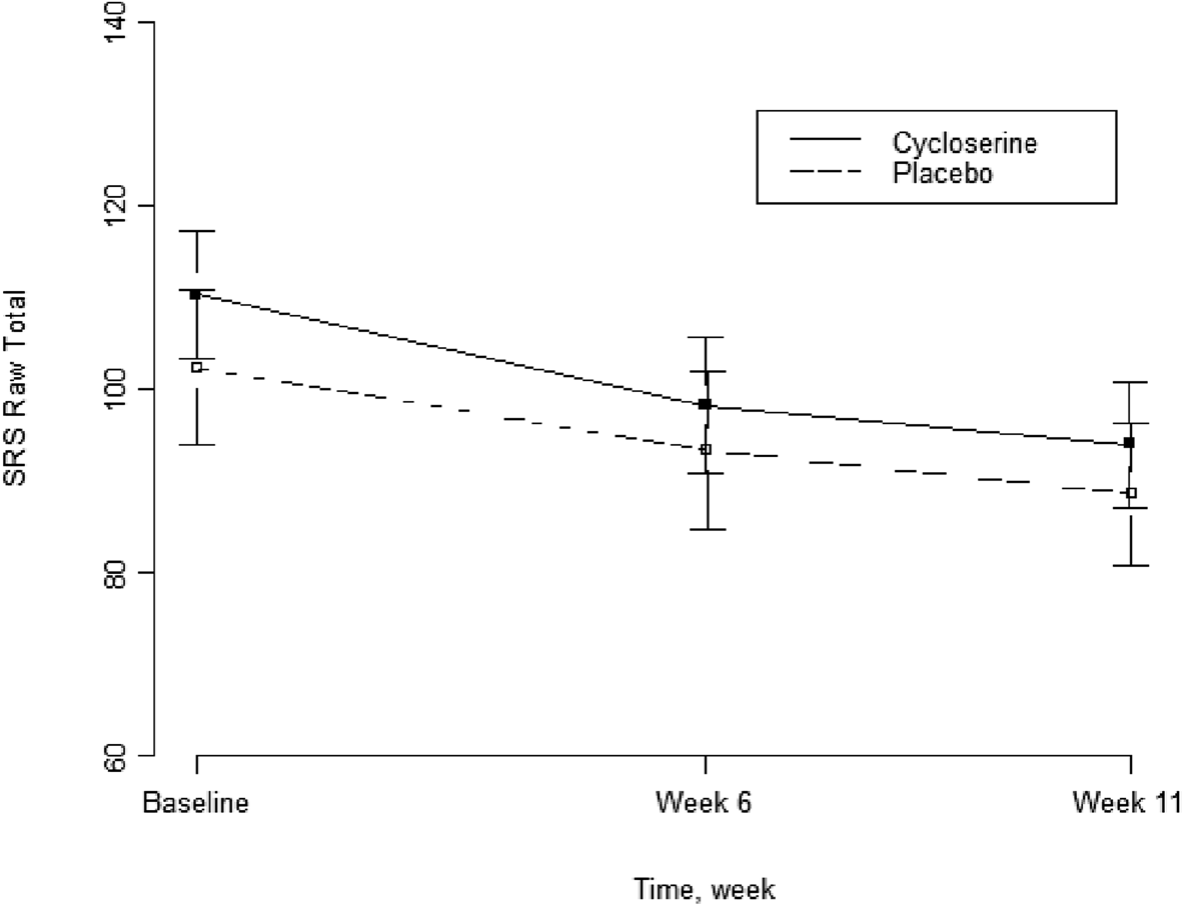
Social responsiveness scale raw score across time points

In addition to the primary endpoint of the study at week 11, the SRS total score was also measured at week 6. A linear mixed-effects model was fitted to further test the treatment effect over time using data at all three visits. Again, there was no significant difference between the two treatment groups (*p* = 0.502). The repeated measures of the SRS total scores are depicted in Fig. 1.

A responder analysis was conducted based on CGI-I scores at 11-week follow-up. For the responder analysis, 33.3 % of participants in the DCS group were classified as responders to treatment based on the CGI-I, as compared to 32.3 % in the placebo group, which showed no significant difference in rate of response between groups (*p* = 0.927). Based on the observed trend of improvement in both treatment groups, subjects were combined to assess whether SRS total score changed significantly from baseline to week 11. A paired *t* test for all 67 subjects with ASD showed a mean change score of −15.14 with 95 % confidence interval (−19.90, −10.38), *p* < 0.0001.

### Adverse events

Table 4 shows the number of subjects who reported an adverse event, as well as all categories of AEs where at least 10 % of either group (DCS or placebo) reported experiencing that AE. Fisher’s exact tests were utilized to derive *p* values. No category of adverse event showed a statistically significant difference between groups. The DCS group experienced more emesis than the placebo group (17.6 vs. 6.1 %, *p* = 0.26). Overall, more patients in the DCS group reported at least one adverse event compared to the placebo group (94.2 vs. 84.8 %), although this difference was not significant (*p* = 0.21). The placebo group had a higher number of total adverse events (149 vs. 138) (*p* = 0.87). Finally, only one serious adverse event (one instance of making a suicidal comment at school when angry) was reported in the placebo group.

**Table 4:** Adverse events by treatment group

	Number (%) of patients reporting		
Adverse Event	DCS (N=34)	Placebo (N=33)	p value
Any Adverse Event	32 (94.1)	28 (84.8)	0.26
Headache (including sinus headache)	9 (26.5)	7 (21.2)	0.80
Nasal congestion or Cold	6 (17.6)	8 (24.2)	0.79
Cough	7 (20.6)	7 (21.2	0.99
Vomiting	6 (17.6)	2 (6.1)	0.29
Aggression	2 (5.9)	5 (15.2)	0.45
Increased motor activity	1 (2.9)	5 (15.2)	0.22
Interrupted sleep/ other sleep problems	3 (8.8)	5 (15.2)	0.73
Irritability (including agitation)	16 (47.1)	15 (45.5)	0.99
Restlessness/Agitation	4 (11.8)	3 (9.1)	0.99
Sadness	5 (14.7)	3 (9.1)	0.73
Sedation/Drowsiness	2 (5.9)	6 (18.2)	0.29
Not otherwise listed	10 (29.4)	12 (36.4)	0.83
Any Serious Adverse Event	0	1 (3.0)	0.99

## Discussion

The core social deficits seen in ASD are severely impairing, and few interventions have been identified to successfully and consistently treat these impairments. Several promising studies have shown DCS to enhance behavioral therapy outcomes in individuals with anxiety disorders, as well as demonstrating potential benefits of DCS treatment in ASD. The present study extended these lines of study by evaluating DCS-mediated enhancement of the learning of social skills in children with ASD. The results of this double-blind placebo-controlled short-term trial demonstrate no drug-related improvement on the primary outcome measure, or any of the secondary outcome measures. However, an overall significant improvement in SRS total raw score was observed from baseline to end of treatment for the entire group of children with ASD.

There are several possible explanations for the lack of pharmacological treatment effect in this study. Notably, the large increase in SRS scores observed in both the DCS and placebo groups is greater than that seen in other intervention studies [45]. This may be explained by the novelty of the social skills training group, something that had previously may have been unavailable to the participants. Therefore, demonstrating the added benefits of DCS to an already high placebo response rate may be very difficult. Also, the SRS was collected immediately following the 10-week trial, which does not allow for the assessment of the durability of these findings.

Another potential explanation for the lack of drug effect may be the heterogeneity of ASD, which makes this a particularly challenging population to study and all the more difficult to find effective pharmacological and behavioral interventions. It is also important to note that since study enrollment ended, the *Diagnostic and Statistical Manual of Mental Disorders*, *Fifth Edition* (DSM-5) [46] has been published with revisions resulting in a new category of diagnosis called autism spectrum disorder, along with the restructuring of diagnostic criteria. However, we do not believe that these diagnostic changes would have influenced the results of the current study.

Another potential reason for the lack of drug effect in the current study is that social interactions, and therefore social deficits, are difficult behaviors to objectively quantify due to the ways in which social behavior changes in different settings and circumstances and over time. This study utilized the parent-rated SRS total raw score to evaluate social deficits in ASD. The SRS provides a global perspective on social deficits in ASD. However, the learning occurring during social skills training may not produce effects sufficiently robust to alter these broad, subjective social skills ratings. In the future, a more direct, objective measurement of social behavior and social interest, such as eye tracking, may be required to capture change in social interaction which occurs at a level not readily observable by caregivers and clinicians.

Several additional factors should be considered in evaluating the findings of the current study. Based on the effective dose of DCS used in the studies of DCS plus therapy for treatment of phobias and social anxiety, all subjects in this trial received 50 mg of DCS regardless of weight [47, 48]. It is possible that higher doses (potentially weight based) may have resulted in greater improvement for the DCS group. However, the phobia study by Ressler et al. [47] demonstrated no difference between 50 and 500 mg doses of DCS, so it is unclear what impact dosage adjustment may have provided [47]. Longer duration and more frequent treatment may also need to be considered. Ten weekly doses of DCS and social skills training may not be sufficient to make robust changes in symptoms of social impairment and extended length of treatment and/or daily dosing may be necessary. In addition, the psychotherapy studies referenced in the development of this protocol dealt with operant conditioning via learned extinction. The current study not only utilized some operant conditioning techniques (such as reinforcement) but also used other learning mechanisms in the training of social skills (such as social learning through modeling and role playing). It is possible that DCS has its greatest influence over learned extinction, and our negative results may reveal the limitation of our employed learning mechanism.

Finally, a limitation of this study is the novelty of the social skills training curriculum and lack of a control for the social skills training group. This curriculum was novel and its effects were previously unknown. In addition, treatment fidelity data was not collected and other standardized behavioral and social skills outcome measures, such as the Social Skills Improvement System [49], or other emotion recognition measures [24] were not utilized. All children enrolled in the study received 10 weeks of social skills training, and statistically significant improvements were seen across the outcome measures when drug and placebo groups were combined. These results may point to the efficacy of this social skills training protocol at improving social outcomes for children with ASD. However, this potential mechanism cannot be confirmed without controlling for other factors that potentially influenced the results, such as maturation, time with trained clinicians, attention, and access to peers. A placebo or waitlist control group should be employed in future studies to evaluate the efficacy of our social skills curriculum.

## Conclusions

The present study provides proof of concept that a large sample study combining medication and social skills training in ASD is feasible. Few studies have been conducted in ASD combining pharmacological and behavioral interventions, despite the common blending of these interventions in clinical settings. Future research on the role of targeted drug treatments in augmenting behavioral interventions in ASD is warranted. Despite the negative result of this short-term drug augmentation analysis, we believe that further work focused on durability of treatment response is needed to assess long-term outcome following initial combination treatment in this and other similar projects. Overall, utilizing targeted drug treatment to facilitate learning and acquisition of skills during therapy in ASD warrants additional investigation. Lessons learned in our study of DCS as a potential augmentation strategy to social skills training lay the groundwork for such work.

## Abbreviations

ASD: autism spectrum disorders
DCS: d-cycloserine
SRS: social responsiveness scale
SB-V: Stanford-Binet 5th edition
VABS-II: Vineland adaptive behavior scale 2nd edition
TSSA: Triad social skills assessment
CGI-S: clinical global impression-severity
TPs: typically-developing peer models
AEs: adverse events
PDD-NOS: pervasive developmental disorder not otherwise specified
ABA: applied behavior analysis
ABC: aberrant behavior checklist
CGI-I: clinical global impression improvement scale
DSM-5: Diagnostic and Statistical Manual of Mental Disorders, Fifth Edition
